# Possible influence of sex on the relationship between dual-task gait costs and cognitive decline in older adults

**DOI:** 10.1371/journal.pone.0317365

**Published:** 2025-01-30

**Authors:** Monica Christova, Shane Fresnoza, Christoph Palli, Wolfgang Staubmann, Bernhard Guggenberger

**Affiliations:** 1 Division of Physiology and Pathophysiology, Otto Loewi Research Center for Vascular Biology, Immunology and Inflammation, Medical University of Graz, Graz, Austria; 2 Institute of Physiotherapy, FH Joanneum University of Applied Sciences, Graz, Austria; 3 Department of Psychology, University of Graz, Graz, Austria; 4 BioTechMed, Graz, Austria; 5 Institute of Health Care and Nursing, FH Joanneum University of Applied Sciences, Graz, Austria; 6 Institute of Dietetics and Nutrition, FH Joanneum University of Applied Sciences, Bad Gleichenberg, Austria; 7 Department of Orthopaedics and Trauma, Medical University of Graz, Graz, Austria; Tokyo Metropolitan Institute of Geriatrics and Gerontology, JAPAN

## Abstract

The impact of cognitive decline in older adults can be evaluated with dual-task gait (DTG) testing in which a cognitive task is performed during walking, leading to increased costs of gait. Previous research demonstrated that higher DTG costs correlate with increasing cognitive deficits and with age. The present study was conducted to explore whether the relationship between the DTG costs and cognitive abilities in older individuals is influenced by sex differences. To address this objective, we conducted a study with 216 elderly participants (age range: 60 to 75 years, 127 females). These underwent Cognitive Functional Dementia (CFD) testing to determine their cognitive abilities and DTG testing to evaluate their gait parameters (gait speed, cadence, stride length, stride variance, and stance phase duration) while performing a backward serial number counting task. We carried out a correlation analysis between the CFD scores and the DTG costs. The DTG costs were calculated as the percentage difference between the gait parameters in single- and in dual-task testing, and the effects were compared considering the factors of sex and age. A significant negative correlation between the CFD scores and the dual-task costs of gait parameters was found only in males. The DTG costs did not differ between the sexes, while women obtained superior scores in the CFD test. The higher DTG costs significantly correlated with older age in men. In summary, our study provides evidence that, unlike in women, the DTG costs during the backward serial number counting task significantly increase in older men, correlating with declines in cognitive performance and increasing age. These findings suggest that the assessment of DT gait characteristics in relation to cognitive decline in older adults may manifest differently between sexes.

## Introduction

Movement and cognition share a common neural substrate, which underlie the relation between our motor and cognitive behaviors. For example, purposeful locomotion is not merely an automatic repetition of steps, but rather a goal-oriented process that requires attention, memory, and executive functions [1,2]. Moreover, cognitive tasks such as memory retrieval, speech perception and production, or reasoning and decision making are commonly performed concurrently with walking. The execution of a secondary cognitive task while walking, which is known as a cognitive motor dual-task (DT), can negatively affect the gait parameters. These DT effects are commonly prescribed to the limited capacity of the cognitive system (e.g., attentional resources), resulting in a bottleneck while processing of two temporally overlapping tasks [3]. The dual- task order regulation in the bottleneck stage is assumed to be maintained and processed in the working memory [4,5]. The working memory is related to the ability to store and manipulate behaviorally useful information for a short time. This ability declines with age and is considered as a core component of aging-associated cognitive deficits [6]. Consequently, the DT gait control decreases in older adults due to their reliance on the working memory resources [7]. For instance, a dual-task involving backward serial number counting while walking, is challenging because the counting task engages the working memory [8], thereby depleting the resources needed for locomotion, (e.g., for motor planning use). Furthermore, the DT performance has been associated with increased activity in the prefrontal, cingulate, parietal, and premotor cortices [9], which are also involved in gait control in older individuals. Higher DT-elicited activation has been observed especially in the prefrontal cortex (PFC) in the elderly, and this activation has been negatively correlated with DT gait [10].

Reduced cognitive status has been associated with gait speed reduction under dual-task conditions using different cognitive tasks [1]. DT walking has been shown to be more closely related to real-world mobility [11] and has been widely used to explore the cognitive demand of gait control [12] in older individuals with preserved cognition [13], as well as in those with mild cognitive impairments (MCIs) [14,15]. When evaluating cognitive status and potential declines, analyses of DT gait have also been recognized as more sensitive than single-task gait analysis [16–18] and are better at detecting subtle changes in gait characteristics in aging populations [19]. To evaluate the magnitude of gait changes during the dual-task performance, the dual-task cost (DTC) is calculated as the percentage difference between the gait parameters in the single-task and the DT [7], with higher DTC indicating more detrimental effect of the cognitive task on gait. Compared to the original gait parameters, which primarily reflect physical abilities, the DTC of gait parameters provides a measure of the detrimental effect of cognitive challenges on gait [20]. Moreover, this calculation reduces the influence of subjective factors such as height, weight, physical health status and activity level [21].

The relationship between the DTC of gait and cognitive declines in an older population can also be influenced by sex-specific differences in cognitive processing [22] and in cognitive aging [23]. In terms of executive functions, males and females have been shown to use different strategies depending on the task demands [24]. Specifically, working memory and attention tasks tend to activate differential neural networks in men and women [25,26].

Sex-related differences in the DTG costs and DTG performance have been examined in younger and older adults by implementing different cognitive paradigms. In a study on younger individuals, dual-task tests that included reaction time-, auditory Stroop-, mental calculation-, memory- and verbal fluency task [27] did not reveal a major influence of gender on motor-cognitive interference during the DT Timed Up and Go Test. In another study, adolescents were required to spell words backwards, subtract numbers and name months in the reverse order. Here, a decreased DTC of walking cadence was revealed in female adolescents with sport-related concussions, but not in the healthy controls [28]. In two other studies, female older participants who were required to walk while counting backwards displayed a higher DTC of gait than males [29,30]. In contrast, females showed better DTG performance with faster step times [31] and lower stride variability while spelling words backwards than their male peers [32].

Despite the sex-related differences in the neurocognitive and motor domains, DT gait testing is commonly implemented to evaluate the cognitive status in older individuals, and sex is disregarded as a possible variable when analyzing and reporting results. The present study was conducted to investigate how aging-related cognitive changes correlate with DT gait changes in older men and women, thus revealing how sex affects this relationship. Prior research clearly shows sex-specific differences in brain morphology and function [33,34], neurocognitive impairments, cognitive aging [35,36], and DTG costs and performance [30–32]. Based on this research, we expect that the correlation between cognitive performance and dual-task gait costs (during backward serial number counting) will differ between men and women as well. Specifically, we hypothesize that the cognitive declines in older males will be better reflected by an increased DTC of gait than in older females, since men exhibit more significant grey matter degeneration in the PFC [37], which is involved in DTG control. Accordingly, we also hypothesize that older males will tend to be more strongly challenged when asked to manage a motor task while concurrently performing a cognitive task. Thus, we anticipate that reduced cognitive performance as assessed with the Cognitive Functions Dementia test will correlate more strongly with increased DTCs of gait velocity and cadence in men than in women.

## Materials and methods

### Participants

The study was conducted on 216 elderly participants ☯median age: 66 years (interquartile range (IQR): 63–70 years), mean age ± SD: 66.70 ± 4.23 years, age range: 60–75 years] comprising 89 males ☯median age: 67 years (IQR: 64–71 years), mean age + SD: 67.57 ± 4.61 years] and 127 females ☯median age: 65 years (IQR: 63–69 years), mean age + SD: 66.10 ± 3.86 years]. The general participant characteristics are presented in Table 1. The participants were recruited using convenience sampling between 1. April and 31.May, 2020. A priori calculation using G*Power 3.1.9.2 indicates that a sample size of 214 (107 participants per group) is sufficient to achieve a statistical power (1-β) of 95% at an alpha level of 0.05 and a large effect size (Cohen’s *q* = 0.50) for correlating two independent Pearson’s r [38]. All had normal or corrected-to-normal vision and were right-handed, according to their self-assessment. Volunteers were not allowed to participate in the study if they met one or more of the following exclusion criteria: clinical diagnosis of MCIs or dementia and psychiatric illness (e.g., depression, psychosis), reduced mobility (walking aid) with gait disturbance, hearing or visual impairment, participation in any other cognitive training study within the last six months, or insufficient competence in the German language. At the beginning of the testing, the participants’ medical history, including past or present neurological, cardiovascular, respiratory, chronic metabolic disorders and pain, was recorded using a questionnaire to ensure that there was no disorder which could affect the gait. Physiological gait characteristics (e.g., absence of limping, symmetric gait) were additionally observed before the experimental session by the test leader. The study was performed following the Helsinki Declaration and was approved by the Medical University of Graz Ethics Committee (EC No. 32–016 ex 19/20). Informed consent was obtained from the participants before the experiment. Eligible participants were recruited by advertising in local newspapers and on social media platforms, contacting pensioners’ clubs, distributing advertisements to the offices of general practitioners, and broadcasting a short report on local television.

**Table 1:** Participant characteristics (mean, SD).

	Total sample (n = 216)	Female (n = 127)	Male (n = 89)
Age (years)	66.7 (4.2)	66.1 (3.9)	67.6 (4.6)
Height (cm)	168.8 (8.8)	163.7 (5.9)	176.0 (6.9)
Weight (kg)	75.4 (14.8)	69.3 (12.2)	84.2 (13.9)
Education (years)	11.93 (3.17)	11.87 (3.04)	12.01 (3.36)

### Experimental design and protocol

This single-center observational study was one of preliminary validation studies performed as part of a larger project (Smart Cognition and Behaviour Screening for early detection of cognitive impairment powered by Augmented Reality). The participants were informed verbally and in writing of the study’s aim and procedure. All data were collected at the JOANNEUM University of Applied Sciences in Graz, Austria. The assessment procedure included the performance of the CFD test set and DT test, performed in a randomized order. The complete assessment was carried out and monitored by trained project staff. In addition, a medical doctor in the role of the clinical investigator was available to provide clarifications if the CFD test results indicate a tendency toward cognitive impairments. All participants were provided with their CFD test results and asked to make an appointment independently if they wanted to discuss the results in detail with the medical doctor. The entire test procedure lasted 80–100 minutes.

### Cognitive functions dementia test set

The Cognitive Functions Dementia (CFD) digital test battery is used to examine the cognitive performance of people older than 50 years, and particularly those with neurodegenerative diseases [39]. The CFD test battery is part of the Wiener Test System NEURO (SCHUHFRIED GmbH, Moedling, Austria) and is a validated tool used to assess cognitive functions [40]. The CFD test battery is used to assess attention, verbal long-term memory, executive functions, and verbal fluency, as well as perceptual motor functions. Parts of the CFD test have been used to evaluate memory performance in patients with heart failure [41], to measure the enhancement of working memory with transcranial direct current stimulation [42], and to predict verbal fluency in healthy persons [43]. The standard version of the test battery includes the following assessments: Vienna Word Fluency Test (WIWO), Auditory Word List Learning Test (AWLT), Perception and Attention Functions Battery–Alertness (WAFA) and Divided attention (WAFG), Trail Making Test–Langensteinbacher Version (TMT), Corsi -block-tapping-test (CORSI) and Vienna Object Naming Test (WOBT) [39]. These tests and how they were applied in this study are described in more details as follows: (1.) The WIWO is used to test the semantic and lexical verbal fluency. The "verbal fluency" was calculated based on the number of correctly named words. (2.) The AWLT was used to test verbal long-term memory by testing the immediate, short-term, and long-term recall of 12 learned words, as well as the recognition of the 12 learned words in a list that also contains 12 new words. (3.) In the WAFA test for alertness, the reaction time to a simple visual stimulus (black circle on a white background) was measured and “averaged”. (4.) To test divided attention (WAFG), the participants received visual and auditory stimuli and were asked to only react when the target stimuli occurred twice in a sequence. The participants’ response times were recorded and averaged. (5.) The TMT tests the ability to process stimuli quickly. It consists of connecting numerical (numbers from 1–25) and alphabetic (letters from A-L) symbols appearing on a screen in an ascending order. Both the speed of task processing and the number of mistakes were recorded. (6.) The Corsi -block-tapping-test is used to assess the spatial working memory capacity and involves sequential tapping on cubes presented on a screen. The study participants had to tap the cubes in a sequence similar to the one given to them before a trial. The number of successful trials was recorded. (7.) The WOBT was used evaluate the participant’s ability to recognize and name objects presented on a screen. The main variable was the number of correct terms.

The evaluation of CFD scores was performed using the standard sample 50+ CFD [44]. Since the CFD test battery consists of the individual test results, the outcome is reported as an overall percentage rank. The weighting of the individual dimensions is based on a theoretically derived and empirically tested structural equation model [39,40]. In our analysis, we used the percentage rank of the CFD index, where a higher percentage indicated better cognitive performance. Based on the CFD index, the cognitive declines are classified as: “no general cognitive impairment” (percentage rank ≥ 16), “suspicion of mild cognitive impairment” (percentage range 4 to 15), or “suspicion of severe cognitive impairment” (percentage rank ≤ 3).

### Dual-task test

The DT test required participants to perform backward serial number counting while walking. In the backward serial number counting task, participants were asked to count backward aloud by threes from 100 (e.g., 100, 97, 94…) while walking on a flat, straight level pathway. Counting backwards was chosen because this task has been shown to involve both attention and working memory [8]. Such internally interfering tasks have a greater impact on changes in gait than those interfering externally such as reaction time tasks [1,2]. Furthermore, serial subtractions were shown to be relevant cognitive tasks for testing older adults [45,46] and to result in consistent changes in DT walking [47–49]. In our study, we asked participants to count backwards by—threes, since counting by—ones appeared to be less demanding and automatic [49], and since counting by sevens was rather frustrating for most participants as observed in our preliminary tests.

**Fig 1:**
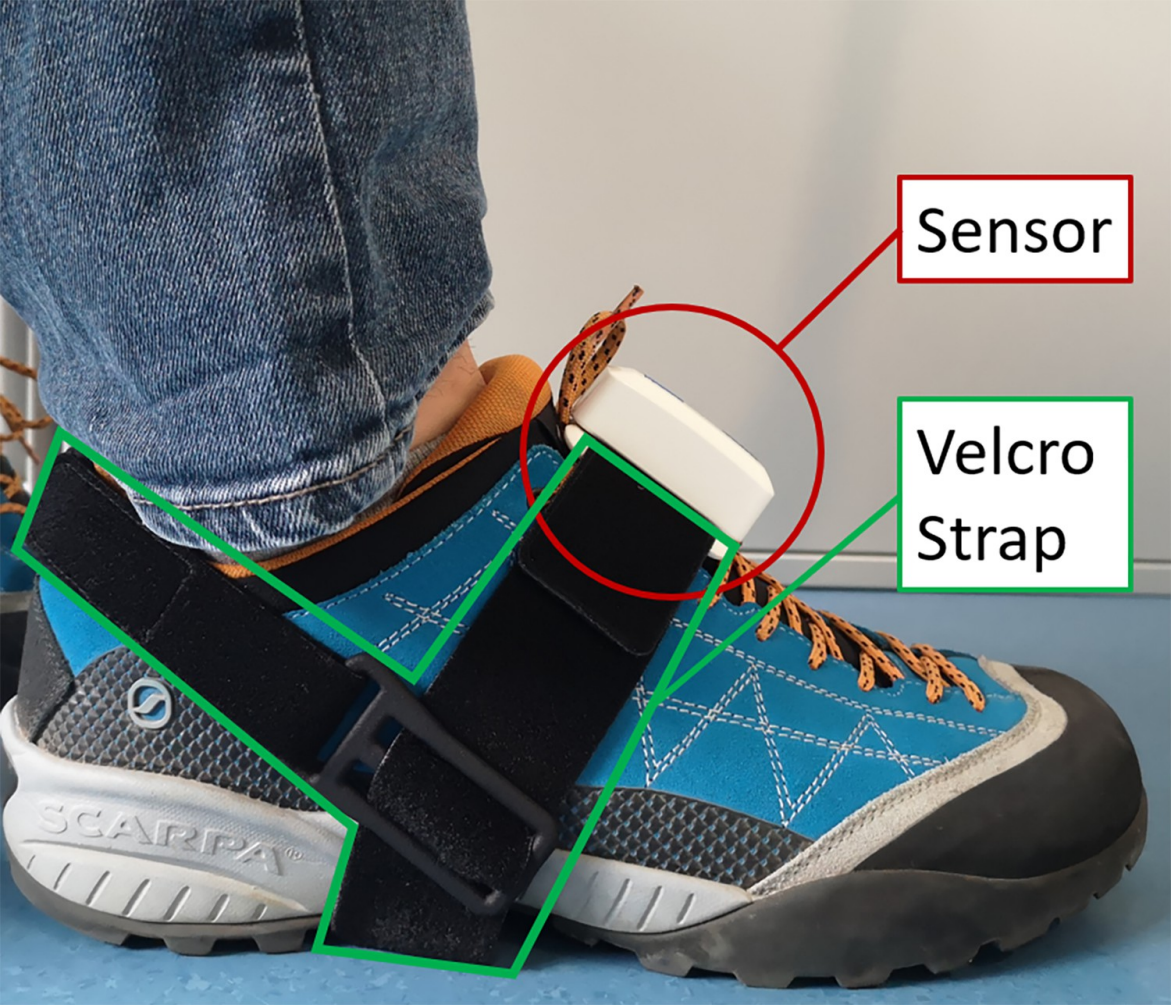
System for evaluation of gait parameters. One sensor was attached with a Velcro strap to the instep of each foot. Each sensor measured three-dimensional linear and rotational acceleration. Using a post-processing algorithm, acceleration data were used to calculate spatiotemporal gait parameters.

The walking distance was set to 20 m to provide enough space for acceleration, walking and deceleration. Participants were instructed to perform both tasks as best as they could, without prioritization. The instruction was to walk at a comfortable self-paced gait speed. Furthermore, they were instructed where to stop. An instrumented gait analysis setup, based on two inertial measurement units (PabloGait, Tyromotion, Graz, Austria) was used for assessing spatial and temporal gait parameters. One inertial measurement unit was placed on the instep of each foot and secured with a Velcro strap system (Fig 1). The walking speed, cadence, stride length, stride variance, and stance phase duration were calculated using a validated calibration-free algorithm [50]. For single-task reference, participants walked the 20 m walkway at a comfortable, self-paced speed. To evaluate DT performance, participants walked the 20m walkway a second time while performing the counting task. To avoid possible learning effects, the two tests were performed once and in a randomized order. To evaluate the outcomes of the DT test, the DTC costs providing the difference in percentage between the single task reference (Eq 1) and the DT performance were calculated for each gait parameter for walking speed, cadence, stride length stride variance and stance phase duration, respectively [7].


$$Dual\;Task\;Cost=\frac{single\;task-dual\;task}{single\;task}*100$$ (1)

### Statistical analysis

The data analyses were performed using SPSS 29 software (IBM Corp., Armonk, NY, USA). Initially, the distribution of demographic data (age, weight and height), CFD score, and dual- task gait costs (gait speed, step frequency, stride length, stride variance, and stance phase) were tested using the Shapiro-Wilk Test. All *p* values were found to be > 0.05, indicating non-normal distributions of the data; hence, to evaluate gender differences, we performed the non-parametric Mann-Whitney U tests on the data. Subsequently, the Spearman’s rank correlation was computed to assess the relationship between CFD and age, as well as between age and each of the DT gait parameter for males and females (separately). Lastly, using an online free software (https://www.psychometrica.de/correlation.html), we tested whether the correlation coefficients (*’ρ’* or *rho*) between the two sexes (e.g., CFD and gait speed rho in females vs CFD and gait rho in males) statistically differ. The level of significance was set at *α* = < .05.

## Results

The CFD scores and DTC of gait results are presented in Table 2. No significant differences were detected regarding the education level and the general health status between men and women. A history of traumatic brain injury was reported by three males and three females, and of stroke,—by one male and one female. The Mann-Whitney U test performed on the demographic data revealed that male participants (median = 67 years, IQR = 64–71 years) were significantly older on average than female (median = 65 years, IQR = 63–69 years) participants, *U* = 6694, *p* = .021. They were also significantly taller (male median = 175 cm, IQR = 171–180 cm; female median = 164 cm IQR = 160–168 cm), *U* = 10416, *p* = < .001; and heavier (male median = 81 kg, IQR = 75–92 kg; female median = 67 kg, IQR = 60.5–75 kg), *U* = 9099, *p* = < .001 than female participants. In terms of CFD, the percent test score was 65.54% ± 26.23 for the female and 52.70% ± 32,66 for the male group, indicating “no general cognitive impairment”. Furthermore, the test revealed that female participants (median = 74%, IQR = 48–85%) had significantly higher cognitive performance than the male participants (median = 54%, IQR = 20–84%), *U* = 4439.50, *p* = .007). Meanwhile, the Mann-Whitney U test comparing the individual DTCs of gait parameters between sexes, revealed non-significant differences (gait speed: *p* = .846, step frequency: *p* = .470, stride length: *p* = .076, stride variance: *p* = .385, and stance phase: *p* = .569) (S1 Table).

**Table 2:** Results from the cognitive function dementia test and the dual-task costs of gait (mean, SD).

Variable	All (n = 216)	Females (n = 127)	Males (n = 89)
CFD index (%)	60.25 (29.66)	65.54 (26.23)	52,70 (32.66)
DTC gait speed	0.16 (0.14)	0.15 (0.14)	0.16 (0.15)
DTC gait frequency	0.12 (0.12)	0.11 (0.12)	0.13 (0.12)
DTC stride length	0.06 (0.09)	0.06 (0.06)	0.06 (0.12)
DTC stride variance	-0.78 (2.11)	-0.70 (2.18)	-0.89 (2.02)
DTC stance phase	-0.02 (0.05)	-0.02 (0.05)	-0.02 (0.06)

Notes: Dual-task costs (DTC), Cognitive Function Dementia test (CFD).

### Relationship between CFD and DTC of gait parameters

**Fig 2:**
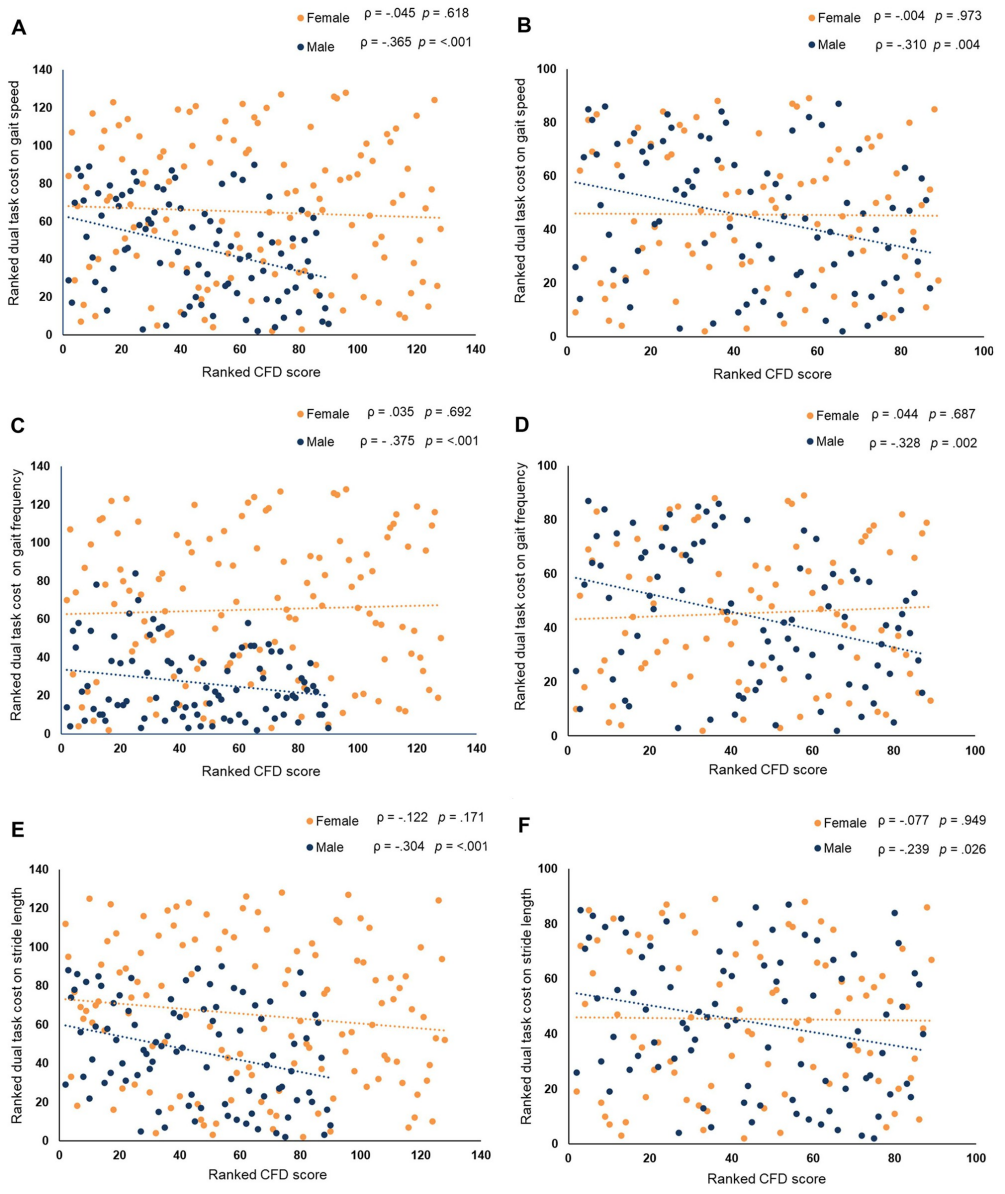
Scatter plots of ranked Cognitive Functions Dementia (CFD) test scores and ranked Dual-Tasking (DT) Cost on gait parameters. (**A** and **B**) Correlation between ranked CFD scores and ranked gait speed. (**C** and **D**) Correlation between ranked CFD scores and gait frequency. (**E** and **F**) Correlation between ranked CFD scores and stride length. The results of Spearman’s rank correlation performed on the complete dataset are shown in the right panels (A, C, and E), and datasets with comparable CFD scores between sexes are shown in the left panels (B, D, and F). Data points in dark blue and orange represent the male and female participants’ data, respectively. Spearman’s rank correlation coefficients (ρ) and corresponding p values are shown above each scatter plot. The dashed lines represent a linear trend.

In female participants, we did not find significant correlations between CFD and DTC of gait parameters: gait speed (*r* (125) = —.045, *p* = .618), gait frequency (*r* (125) = .035, *p* = .692), stride length (*r* (125) = —.122, *p* = .171), stride variance (*r* (125) = .020, *p* = .826), and stance phase (*r* (125) = .020, *p* = .772). In contrast, in male participants, we found significant negative correlations between CFD and DTCs of gait parameters: gait speed (*r* (87) = —.365, *p* = < .001), gait frequency (*r* (87) = —.375, *p* = < .001), and stride length (*r* (87) = —.304, *p* = .004) (Fig 2A, 2C and 2E). These results indicate that the DTCs of gait speed, gait frequency, and stride length are high when cognitive performance in male participants is low or vice versa. On the other hand, the positive correlations between CFD and DTC of stride variance (*r* (87) = .196, *p* = .066) and CFD and DTC of stance phase (*r* (87) = .170, *p* = .110) in male participants were not significant. Comparisons of the correlation coefficients between sexes showed significantly stronger correlations for CFD and DTC of gait speed (*z* = 2.41, *p* = .008), and CFD and DTC of gait frequency (*z* = 3.06, *p* = .001) in males than in females. The correlation coefficients of CFD and DTCs of gait parameters: stride variance (*z* = -1.27, *p* = .102), stride length (*z* = 1.36, *p* = .086), and stance phase (z = -1.08, *p* = .140) were comparable between both groups.

The significant correlations between CFD and the DTCs of gait variables (gait speed, gait frequency, and stride length) in males and the absence of any correlations in females could have been influenced by the robust differences in cognitive skills (as measured by CFD) between the groups (male median = 54%; female median = 74%). In this case, female participants significantly outperformed the male participants. Therefore, we repeated the correlational analysis in both groups using comparable mean CFD (male = 54.43%; female median = 54.77%). The comparable between-group CFD was achieved by excluding participants with extreme values: in the female group, 38 with CFD > 83% and 1 with CFD = 0 (n = 39), and in the male group 3 participants with CFD = 0. The pattern of results was similar to that of the full dataset. In female participants (Fig 2B, 2D and 2F), we again did not find significant correlations between CFD and the DTC of gait parameters: gait speed (*r* (87) = —.004, *p* = .973), gait frequency (*r* (87) = .044, *p* = .687), stride length (*r* (87) = —.077, *p* = .949), stride variance (*r* (87) = .062, *p* = .567) and stance phase (*r* (87) = .015, *p* = .889). For the male participants (Fig 2B, 2D and 2F), significant negative correlations were again observed between CFD and DTCs of gait speed (*r* (85) = —.310, *p* = .004), gait frequency (*r* (85) = —.328, *p* = .002), and stride length (*r* (85) = —.239, *p* = .026). Also, the positive correlations between CFD and the DTCs of stride variance (*r* (85) = .132, *p* = .227) and stance phase (*r* (85) = .123, *p* = .261) did not reach significance. In comparing correlation coefficients between groups, significantly stronger correlations for CFD and the DTC of gait speed (*z* = -2.09, *p* = .018) and CFD and DTC of gait frequency (*z* = 2.48, *p* = .007) were again observed in males than in females. The correlation coefficients of CFD and DTC for: stride variance (*z* = -0.46, *p* = .324), stride length (*z* = 1.07, *p* = .142), and stance phase (*z* = -0.7, *p* = .242) were comparable between genders.

### Relationship between age and DTCs of gait parameters and CFD

In terms of age, we found a significant negative correlation between the female participant’s age and CFD score (*r* (127) = —.387, *p* = < .001), indicating a decrease in cognitive performance with increasing age. The correlations between the female participants’ age and DTC of gait speed (*r* (127) = —.115, *p* = .197), gait frequency (*r* (127) = —.168, *p* = .058), stride length (*r* (127) = —.020, *p* = .827), stance phase (*r* (127) = -.046, *p* = .610), and stride variance (*r* (127) = .078, *p* = .381) did not reach significance.

Regarding the male participants, their ages significantly correlated with their cognitive performance in terms of the CFD score (*r* (89) = —.417, *p* = < .001). This negative correlation also means that as their age increases, their cognitive performance decreases. With respect to the DTC of gait parameters, the male participants’ ages significantly correlated with the gait speed (*r* (89) = .215, *p* = .042), indicating that their DTC of gait speed increases as they age. Similarly, their age significantly (and positively) correlated with the DTC of gait frequency (*r* (89) = .217, *p* = .041), meaning that the DTC of gait frequency increases as their age increases. Meanwhile, the correlations between their age and the DTC of stride length, (*r* (89) = .170, *p* = .111), stride variance (*r* (89) = -.184, *p* = .084), and stance phase (*r* (89) = -.083, *p* = .441) remained non-significant. The comparisons of correlation coefficients showed that males have stronger correlations than females. This was significant regarding the differences seen for age and the DTC of gait speed (*z* = -2.38, *p* = .009), age and the DTC of gait frequency (*z* = -2.78, *p* = .003), and age and the DTC of stride variance (*z* = 1.883, *p* = .030). This was, however, not significant for age and CFD (*z* = -.225, *p* = .399), age and DTC of stride length (*z* = -1.366, *p* = .086), and age and DTC of stance phase (*z* = .265, *p* = .396).

The significant correlations between age and the DTCs of gait variables, and specifically gait speed and gait frequency in males, may have been influenced by the age differences between the sexes, because the males were significantly older than females. To rule this out, we also repeated the correlational analysis in both groups using comparable ages (male = 67.27 years; female = 66.30 years) achieved by excluding two male participants, ages 80 and 81 years, and four 60-year-old female participants. Like the initial results, we saw a significant negative correlation between the ages and CFD scores in both groups (female: *r* (123) = -.370, *p* = < .001; male: *r* (87) = -.432, *p* = < .001) indicating cognitive performance reduction in both groups with comparable increasing age. For the correlation of age with DTC of gait parameters, the new findings include the significant negative correlation between age and gait frequency in females (*r* (123) = —.190, *p* = .035) and between age and stride variance in males (*r* (87) = —.250, *p* = .019). Meanwhile, the correlation coefficient comparisons between the groups with balanced age revealed the same pattern of results as in the initial comparisons: The male group displayed higher correlations than the female group, (see Table 3). Like the original analysis, the differences in the correlation coefficient were only significant for age and DTC of gait speed (*z* = -2.519, *p* = .006), age and DTC of gait frequency (*z* = -3.125, *p* = .001), and age and DTC of stride variance (*z* = 2.324, *p* = .01). The differences in the correlation coefficient for age and CFD score (*z* = 0.52, *p* = .302), age and DTC of stride length (z = -1.374, *p* = .085), and age and DTC of stance phase (z = 0.353, *p* = .362) remained not significant.

**Table 3:** Results for Spearman rank correlation coefficients (Spearman’s rho) for CFD, age, weight and height and dual-task cost for gait parameters.

	Raw data				Balanced data			
*CFD*								
	Female (65.54 ± 26.23%)		Male (52.69 ± 32.65%)		Female (54.53 ± 31.66%)		Male (54.30 ± 22.54%)	
*Correlation with*:	r value	*p-value*	r value	*p-value*	r value	*p-value*	r value	*p-value*
Gait speed	-.045	.618	-.365	< .001*	-.004	.973	-.310	.004*
Gait frequency	.035	.692	-.375	< .001*	.044	.687	-.328	.002*
Stride length	-.122	.171	-.304	.004*	-.007	.949	-.239	.026*
Stride variance	.020	.826	.196	.066	.062	.567	.132	.227
Stance phase	.020	.772	.170	.110	.015	.889	.123	.261
*Age*								
	Female (66.10 ± 3.88 yrs)		Male (67.57 ± 4.61 yrs)		Female (66.30 ± 3.76 yrs)		Male (67.27 ± 4.22 yrs)	
*Correlation with*:	r value	*p-value*	r value	*p-value*	r value	*p-value*	r value	*p-value*
CFD	-.387	< .001*	-.417	< .001*	-.370	< .001*	-.432	< .001*
Gait speed	-.115	.197	.215	.042*	-.138	.127	.236	.028*
Gait frequency	-.168	.058	.217	.041*	-.190	.035*	.247	.021*
Stride length	-.020	.827	.170	.111	-.032	.723	.162	.134
Stride variance	.078	.381	-.184	.084	.075	.413	-.250	.019*
Stance phase	-.046	.610	-.083	.441	-.034	.706	-.084	.437
*Weight*								
	Female (69.29 ± 12.20 kg)		Male (84.15 ± 13.86 kg)		Female (78.80 ± 9.62 kg)		Male (79.85 ± 9.17 kg)	
*Correlation with*:	r value	*p-value*	r value	*p-value*	r value	*p-value*	r value	*p-value*
CFD	.252	.004*	-.094	.382	.206	.100	-.117	.315
Gait speed	.016	.862	.007	.947	.138	.274	.091	.433
Gait frequency	.039	.661	.085	.430	.151	.231	.102	.380
Stride length	-.067	.452	-.040	.712	-.006	.961	.130	.263
Stride variance	.066	.458	-.144	.177	-.027	.832	-.085	.466
Stance phase	-.003	.978	.050	.645	-.093	.461	-.071	.545
*Height*								
	Female (163.71 ± 5.91 cm)		Male (175.99 ± 6.89 cm)		Female (169.36 ± 3.96 cm)		Male (171.55 ± 3.71 cm)	
*Correlation with*:	r value	*p-value*	r value	*p-value*	r value	*p-value*	r value	*p-value*
CFD	.220	.013*	.058	.589	.440	.002*	.155	.257
Gait speed	-.007	.936	-.047	.661	.094	.536	-.050	.719
Gait frequency	.115	.198	-.031	.772	.109	.471	.004	.977
Stride length	-.167	.060	-.006	.953	-.044	.772	-.022	.871
Stride variance	.018	.844	-.061	.569	-.173	.251	-.121	.379
Stance phase	.102	.253	-.101	.346	.101	.503	-.080	.559

Note: *Significant level at alpha 0.05 Cognitive Function Dementia test (CFD), Dual-task costs (DTC).

## Discussion

The present study was designed to examine whether the cognitive abilities of older male and female adults correlate with the DTC of their gait parameters during backward serial number counting. We found significant negative correlations between the cognitive performance as measured with CFD and the DTCs of gait parameters in male participants, while such a correlation was not found in females. Males and females exhibited comparable DTCs of gait, while females showed CFD performance that was superior to males. The results also show that increasing age correlated negatively with cognitive performance (CFD score) in both male and female participants; however, only the males’ age significantly correlated with the DTC of gait parameters.

### Effect of sex on the relationship between the cognitive performance and DTC of gait

There is substantial evidence that higher DT gait costs are associated with greater cognitive decline [51,52]. In our study, we observed these effects only in males who showed lower cognitive scores in the CFD test and higher DTC of gait parameters, expressed as slower walking speed, decreased gait frequency, and reduced stride length. These findings can be attributed to the differential involvement of the prefrontal cortical areas in cognitive performance and DT walking in aging. Specifically, the left lateral PFC has been identified as a key functional region subserving the dual-tasking [53]. A gradual increase in PFC activity associated with poorer DT walking in older individuals, has been shown in fNIRS studies [10]. Activation in the bilateral PFC, left posterior inferior parietal lobule, and left supplementary motor area has been observed during backward number counting [54,55]. In another study, higher PFC activity indicated that increased cognitive efforts were necessary to complete tasks, and this could be considered as a marker of limited cognitive capacity [56]. Furthermore, PFC has been shown to be involved in cognitive declines associated with detrimental connectivity alterations and grey matter atrophy in this region [57]. Previous research showed sex-specific differences in PFC structural and functional organization. For example, higher oxygenated hemoglobin levels were recorded in men than in women during attention-demanding locomotion tasks in PFC [58]. This result suggests that men must invest higher cognitive efforts to complete the walking task, which respectively leads to an increased DTC of gait if these cognitive demands cannot be fulfilled. Men tend to exhibit a reduced volume of the frontal lobes [59], more significant grey matter degeneration in the PFC [37], and lower fractional anisotropy in the left frontal lobe [60] than women. These detriments may result in disruption of the related neural networks necessary for DT walking in men, thereby increasing the gait costs when the cognitive reserves are reduced. Lastly, healthy aging women show higher connectivity in the default mode network [61,62] which can apparently compensate for the aging-related global and regional neuronal loss, thus accounting for better DTG performance and lower DTG costs, even in the presence of cognitive deficits.

### Effect of sex on cognitive performance

In the CFD test evaluating the cognitive functions, women showed significantly better performance than men. This result could be prescribed to the fact that the female group in our sample was younger than the male group, as advancing age is associated with declining cognitive functions. Repeating the analysis with age balanced between sexes revealed that, even at a comparable age, the cognitive decline was more pronounced in male individuals. Furthermore, since the participating women were overall lighter and shorter than the men, which can influence the cognitive performance as well, we performed additional analysis which showed the effects of weight and height on CFD performance in men and in women to be generally insignificant (see Table 3). Moreover, sex was shown to have no significant interaction when using models that assessed the association between older -age, height, and intelligence [63]. It is well known that women have an advantage in language processing in cognitive tests (i.e., phonological discrimination, verbal attention, lexical decision making), while men are superior in visuospatial processing (i.e., mental rotation, visuospatial attention) [22]. These specificities can be attributed to sex-related differences in the organization of the neural networks, as shown in MRI and EEG studies. While the male brain shows more pronounced intra-hemispheric connectivity, modularity and transitivity, and a larger parietal area, the female brain is characterized by better inter-hemispheric connectivity and higher volume of the perisylvian language cortex [34,64,65]. Accordingly, greater focal intra-hemispheric activation has been found in males during spatial task execution [33], and more pronounced inter-hemispheric activation has been found in females while performing a language task [66]. Nevertheless, the verbal advantage of women is unlikely to account for the superior cognitive performance in females in our study, since the implemented CFD test battery combines different tasks containing words, numbers and objects. The level of cognition in older adults is influenced by diverse factors, including the educational level, social activity level, and the number of social connections [67]. In general, women show larger and more varied social networks with more friends and more social support, while men tend to maintain relationships with fewer people [68,69]. Nonetheless, since we did not track the social activities and neuropsychological profile of our participants, we cannot draw clear conclusions regarding the contribution of these factors to our results. Finally, the higher performance in CFD in women was unlikely to account for the absence of a significant correlation between the DTC of gait and the cognitive level in women, because these results remained unchanged even after performing the additional analysis with comparable CFD scores for both sexes.

### Effect of sex on the DTC of gait

The results of our study reveal a nonsignificant effect of sex on the DTC of gait parameters, which partially contrasts with previous findings. For example, higher DTC of gait speed, cadence and double support time were reported in women compared to men when using backward number counting as a cognitive task [29,30,70]. In contrast, other studies implementing spelling words backwards in their DT paradigm and found lower DTC of gait in women as indicated by their faster step times [31] and lower variability during DT than in men [32]. The latter study found no differences in the DTCs of gait speed for both sexes, which was confirmed by our results. The nature and difficulty of the cognitive paradigm chosen to be performed concurrently with the walking task may account for the different results. It has been suggested that women surpass men when performing tasks involving the verbal working memory and verbal attention, while men do better when performing visuospatial processing tasks (for a review, see Ramos-Loyo et al. [22]. Hence, it can be assumed that women show fewer costs of DTG because they perform tasks involving verbal processing with less difficulties (e.g., spelling words). Men, respectively, would have lower DTCs of gait when performing DT involving number-counting tasks; however, this was not supported by results.

Other methodological considerations, such as the provided instructions for task prioritization may also influence the DTCs of gait. In our study, participants were not given specific instructions regarding the prioritization of motor versus cognitive tasks. Consequently, the similar DTC of gait costs could be attributed to both older males and females automatically focusing on the gait task as ‘posture-first’ strategy to maintain balance during walking [71]. This aligns with previous studies that did not implement prioritization instructions and reported no significant differences in DT gait speed in older adults [32], as well as negligible sex-specific differences in the DTC of gait among younger adults [27,28]. Therefore, instruction methods for task prioritization should be carefully considered when evaluating DTCs across different age groups and sexes.

### Effect of age on cognitive performance and on DTC of gait in men and women

The present study revealed the influence of age on the cognitive performance (CFD score) and on the DTC of gait variables, as well as sex-specific correlations between age and DTC of gait parameters. Both men and women showed a similar significant correlation between age and CFD, indicating that the cognitive abilities reduce as age increases regardless of sex. These results are in line with a substantial amount of evidence on aging-related decline in processing speed, attention, memory, language, visuospatial abilities, and executive functions and are underlined by respective functional and structural brain changes [72].

Compared to women, men showed a significantly higher correlation between the age and the changes in the DTC of gait variables. Specifically, significant positive correlations between age and DTC of gait speed and gait frequency were found in men. This indicates that, as their age increases, men walk at a slower velocity and lower frequency during the DT compared to the single-task gait performance. Our findings support those of previous research, which showed significant effect of aging on increased DTC of gait parameters only in men [29]. This effect can be linked to specific parallel age-related changes in cognitive and motor control [73] in men and women. For example, more pronounced effects of age were revealed in the frontal lobes in men [59], while reduced frontoparietal regions have been linked to shorter steps and longer double supporting time [74]. Moreover, an increased DTC of gait speed in older age indicates a higher involvement of cortical attention processes [20]; thus, the increased DTC of gait speed in men may reflect their attenuated attentional resources [75]. These assumptions need to be tested in future studies implementing imaging techniques.

### Limitations and future directions

The present study has certain limitations, and the results should be interpreted in light with them. First, we did not examine the cognitive performance in the dual-task testing, since we were primarily interested in the DT gait variables and their relationship to the cognitive abilities in men and women. Measuring the DTC of the cognitive task can provide additional important information about the role of the prioritization of the DT cognitive versus the DT motor domain in men and women, as shown by Yogev et al. [76] and could better highlight the possible sex- specific effects in our study. Therefore, we can interpret our results only in light of the DT gait variables but not of the DT performance. Second, we have implemented an arithmetic-based task in the DT paradigm. Although this task engages working memory [1], substantially activates prefrontal, parietal, and supplementary areas [54,55], and appears relevant as dual-task protocol for testing older adults [45,46], it might impose a specific type of cognitive challenge on the participants. Therefore, we cannot generalize our results to other DT cognitive paradigms that involve different types of attention and memory. Moreover, the cognitive domain of the DT test does not directly align with the cognitive domains assessed by the CFD, which could be related to the absent correlation between the DTC of gait and the CFD performance in women. In addition, serial subtraction is rarely performed in daily life circumstances. Thus, it does not adequately reflect the more context-dependent cognitive demands in real-world situations, which involve different types of information processing. Understanding the role of cognitive task nature and ecological validity in designing DT protocols to assess cognitive declines in men and women warrants further research.

A further limitation concerns the participants’ characteristics; we did not consider other demographic factors which may influence cognitive declines in older age (e.g., recording nutritional deficiency, physical activity level, and social engagement). A more comprehensive evaluation of participants-specific factors related to cognitive status would help identify more specific links between these variables in relation to sex and cognitive changes. Although our results did not reveal significant effects of body weight and height on cognitive performance, obesity is considered to be a risk factor for cognitive declines [77,78], and a relationship between height and cognitive performance in late adulthood has been demonstrated [79,80]. Therefore, controlling for demographic and participant-specific factors that influence cognitive status should be considered both when recruiting older participants and in analyzing data.

## Conclusions

In the present study, we found that cognitive decline in older adults, as measured by the Cognitive Functional Dementia test, is more strongly associated with dual-task gait costs in males than in females. Specifically, older men exhibited a significant negative correlation between cognitive performance and DTC of gait speed and frequency when performing the dual task, whereas this relationship was not observed in women. Our findings extend those of previous research and provide additional evidence for possible sex-specific interactions between cognitive and gait control. Future studies could explore differences between men and women when using dual-task gait testing to diagnose mild cognitive impairment and dementia.

## Supporting information

S1 Table The non-parametric Mann-Whitney U test results comparing the male and female group’s demographic data, CFD scores, and dual-task cost of gait parameters. (DOCX)
