# Supplementary material for: Possible influence of sex on the relationship between dual-task gait costs and cognitive decline in older adults
Source: PLoS One. 2025 Jan 30;20(1):e0317365. doi: 10.1371/journal.pone.0317365 (PMC11781657; doi:10.1371/journal.pone.0317365)
Supplement: S1 Table — (DOCX) [file pone.0317365.s001.docx]

**Supplementary Table 1.** The non-parametric Mann-Whitney U test results comparing the male and female group’s demographic data, CFD scores, and dual-task cost of gait parameters.

|  | **Female group** | | **Male group** | | **Mann-Whitney U Test** | |
| --- | --- | --- | --- | --- | --- | --- |
|  | Median | IQR | Median | IQR | U-value | *p*-value |
| Age (years) | 65 | 63 - 69 | 67 | 64 - 71 | 6694 | .021* |
| Height (cm) | 164 | 160 - 168 | 175 | 171 - 180 | 10416 | <.001* |
| Weight (kg) | 67 | 60.5 - 75 | 81 | 75 - 92 | 9099 | <.001* |
| CFD (%) | 74 | 48 - 85 | 54 | 20 - 84 | 4439.50 | .007* |
| DTC: |  |  |  |  |  |  |
| gait speed | 0.14 | 0.07 - 0.23 | 0.13 | 0.07 - 0.23 | 5564 | .846 |
| step frequency | 0.09 | 0.04 - 0.145 | 0.1 | 0.04 - 0.16 | 5977.5 | .470 |
| stride length | 0.06 | 0.02 - 0.1 | 0.04 | -0.01 - 0.08 | 4850 | .076 |
| stride variance | -0.2 | -0.5 - 0 | -0.25 | -1 - 0.14 | 5259.5 | .385 |
| stance phase | -0.01 | -0.03 - 0 | -0.01 | -0.03 - 0 | 5906 | .569 |

Note: *Mean Rank differences are significant at the 0.05 level, Cognitive Function Dementia test (CFD), Dual-task costs (DTC)
